# Enduring Personality Changes after Intense Stressful Event: Case Report

**DOI:** 10.3889/oamjms.2016.083

**Published:** 2016-08-17

**Authors:** Slavica Arsova, Nensi Manusheva, Gabriela Kopacheva-Barsova, Stojan Bajraktarov

**Affiliations:** 1 *University Psychiatry Clinic, Faculty of Medicine, Ss Cyril and Methodius University of Skopje, Skopje, Republic of Macedonia*; 2 *University Clinic for Ear, Nose and Throat, Faculty of Medicine, Ss Cyril and Methodius University of Skopje, Vodnjanska 17, Skopje 1109, Republic of Macedonia*

**Keywords:** posttraumatic stress disorder, enduring personality changes, SSRI anti-depressive, anti-psychotic, psycho stabilising therapy, case report, Republic of Macedonia

## Abstract

**BACKGROUND:**

World statistical data show that a large number of individuals suffer from posttraumatic stress disorder (PTSD) after exposure to the intense traumatic event. PTSD can have a chronic course with enduring changes in the functioning of the person.

**CASE PRESENTATION:**

Here we report two adult individuals of different gender and education who were exposed to the extremely severe stressful event after which difficulties in psychological functioning developed. The first case we present is a 46-year-old man, with completed high education, divorced, father of two children, who lives with his parents, and is retired. Disorders appeared 20 years ago when he was exposed to extremely severe stressful events in war circumstances that included captivity, torture, and loss of fellow soldiers. The second case is a 50-year-old female patient, with a university degree, professor of art, married, and mother of two children of whom the son died six years ago. She suffered from disorders after the sudden injury of her son that ended with his death.

**CONCLUSION:**

Posttraumatic stress disorder after the intense stress is a risk of development enduring personality changes with serious individual and social consequences.

## Introduction

World statistical data show that a large number of individuals suffer from posttraumatic stress disorder (PTSD) after exposure to the intense traumatic event. PTSD can have a chronic course with enduring changes in functioning of the person [1].

It is considered that the majority of healthy individuals will not develop posttraumatic stress disorder; however, if the individual has experienced an extremely traumatizing event associated with violence and conflict situations such as war, concentration camp, rape, domestic violence, bullying during childhood, then the chances for development of this disorder are greater [2-6].

Literature data show that men tend to experience more traumatic events, but women more often develop stress-related disorder if they have been exposed to traumatic events such as family violence, loss of a child, rape, etc [7, 8].

According to ICD 10, if psychological difficulties last for years after the survived traumatic event maladaptive forms of behaviour develop including distinct difficulties in social and personal functioning that lead to enduring personality changes [9].

The aim of this study was to present the influence of intense stressful event on the development of enduring personality changes after survived catastrophic event.

## Case Presentation

Here we report two adult individuals of different gender and education who were exposed to the extremely severe stressful event after which difficulties in psychological functioning developed. The diagnosis was made in line with the ICD 10, and the following diagnostic criteria were used: HAMA, HAMD, and clinical interview, psychological and social findings.

The first case we present is a 46-year-old man, with completed high education, divorced, father of two children, who lives with his parents, and is retired. Disorders appeared 20 years ago when he was exposed to extremely severe stressful events in war circumstances that included captivity, torture, and loss of fellow soldiers. Within the course of the first years he developed posttraumatic stress disorder diagnosed and managed on an outpatient basis, and later several hospital stays in day hospital care have been recorded. Variable psychological difficulties with the gradual development of enduring personality changes have been observed along with a persistent feeling of insecurity, jeopardy, social inequality, withdrawal and marked work-related dysfunction.

The second case is a 50-year-old female patient, with a university degree, professor of art, married, and mother of two children of whom the son died six years ago. She suffered from disorders after the sudden injury of her son that ended with his death. Within the course of the first month’s acute stress reaction was noticed, which was later transformed into a posttraumatic stress disorder of chronic course and development of enduring personality changes.

During the day hospital stay patients were treated with pharmacologic agents (SSRI anti-depressive, anti-psychotic and psycho stabilising therapy), which showed a modest success, that is, reduction in the impulsive behaviour; sleep improvement and reduction in self-harmful behaviour. In addition to pharmacology therapy patients were treated with individual and group psychosocial therapy procedures but without significant success in their normal reintegration in their families, social and professional environment.

## Discussion

Literature shows that PTSD most commonly develops in individuals who have been exposed to severe stress associated with violence when the lives of the victims or their close relatives or friends have been in jeopardy, or they have been threatened, tortured, molested, or suffered a sudden loss of a close family member or friend. Long-lasting difficulties might result in permanent maladaptive forms of behaviour [10]. These individuals have paranoid attitude towards the environment, a feeling of uncertainty, social withdrawal, work-related dysfunction, irritability, and they are prone to interpersonal conflicts, with a low threshold of tolerance for frustrating situations [1].

In conclusion, posttraumatic stress disorder after the intense stress is a risk of development enduring personality changes with serious individual and social consequences.
